# Genome Sequence of a *Mycoplasma meleagridis* Field Strain

**DOI:** 10.1128/genomeA.00017-16

**Published:** 2016-03-03

**Authors:** Ticiana S. Rocha, Luigi Bertolotti, Salvatore Catania, Philippe Pourquier, Sergio Rosati

**Affiliations:** a Department of Veterinary Science, University of Turin, Turin, Italy; b Department of Animal Pathology and Public Health, Avian Medicine and Mycoplasma Group, Istituto Zooprofilattico Sperimentale delle Venezie, Padova, Italy; c IDVet-Innovative Diagnostics, Montpellier, France

## Abstract

*Mycoplasma meleagridis* is a major cause of disease and economic loss in turkeys. Here, we report the genome sequence of an *M. meleagridis* field strain, which enlarges the knowledge about this bacterium and helps the identification of possible coding sequences for drug resistance genes and specific antigens.

## GENOME ANNOUNCEMENT

*Mycoplasma meleagridis* is widespread in turkey flocks, causing poor growth, air sacculitis, osteodystrophy, and immunosuppression (1, 2). A recent work provided the first evidence that *M. meleagridis* could also establish a natural infection in chickens (3). Moreover, a number of antigens appear to be shared between *M. meleagridis* and the more important poultry mycoplasmas (*Mycoplasma gallisepticum* and *Mycoplasma synoviae*) resulting in cross-reactivity that complicates serological investigations (2).

The *M. meleagridis* strain used in this study was isolated in 2011 at the Istituto Zooprofilattico Sperimentale delle Venezie, Italy, from a turkey with typical mycoplasma symptoms, including skeletal alterations, by using traditional microbiological methods and denaturing gradient gel electrophoresis-PCR (DGGE-PCR) for confirmation.

The *M. meleagridis* genome was sequenced by using paired-ends Illumina MiSeq technology for 600 cycles and resulted in a total of 27,001,860 reads. ABySS assembly showed the best compromise to be Kmer equal to 63, resulting in 157 scaffolds and good assembly performance (total genome length, 647,259 bp; maximum scaffold length, 181,832 bp; *N*_50_, 90,728 bp; number of scaffolds covering the *N*_50_, 3 scaffolds). Nine of the scaffolds were >1 kb and used for the further analyses. The average coverage of reads on each scaffold was around 8,000×. The genome annotation was using RAST/SEED software and manually reviewed. Five hundred fifty-one putative open reading frames (ORFs) were identified: 513 of them were coding sequences, and 38 were tRNA genes. The metabolic pathways were constructed using the Kyoto Encyclopedia of Genes and Genomes (KEGG) database.

As a result, a total of 276 coding sequences have been assigned a putative functional identity. Two genes were identified as YjeE, which are predicted to have essential role in cell wall biosynthesis; another 16 genes were responsible for membrane transport; 15 genes code for amino acids and derivatives; and 132 genes were responsible for protein metabolism, including two responsible for lipoprotein biosynthesis, which represents important information for future studies regarding possible specific antigens for *M. meleagridis* diagnostics.

Also, four genes for fluoroquinolone resistance (*parcC*, *parcE*, *gyrA*, and *gyrB*) were identified and determined to be active together with one multidrug resistance gene belonging to the multidrug and toxic compound extrusion (MATE) superfamily. Their sequences were subjected to a BLAST search against the previously sequenced genome of an *M. meleagridis* reference strain (4), and we observed some mismatches in the *gyrA* and multidrug resistance genes. This feature is important since we are working with a sample field, and mutations in defined regions of the DNA gyrase genes, *gyrA* and *gyrB*, and the topoisomerase IV genes, *parC* and *parE*, have been linked to high-level fluoroquinolone resistance in various bacteria, including *Neisseria gonorrhoeae* and *Mycoplasma genitalium* (5).

The availability of high-quality genome sequences for an *M. meleagridis* field strain and comparative analyses with related species will improve our understanding of the genes encoding antibiotic resistance and immunodominant antigens for this bacteria that until today have not yet been well characterized.

### Nucleotide sequence accession numbers.

This whole-genome shotgun project has been deposited at DDBJ/EMBL/GenBank under the accession no. LOHQ00000000. The version described in this paper is version LOHQ01000000.
